# Occipital peripheral nerve stimulation in the management of chronic intractable occipital neuralgia in a patient with neurofibromatosis type 1: a case report

**DOI:** 10.1186/1752-1947-5-174

**Published:** 2011-05-10

**Authors:** Ioannis Skaribas, Octavio Calvillo, Evangelia Delikanaki-Skaribas

**Affiliations:** 1 Greater Houston Pain Consultants, Greater Houston Anesthesiology, 2411 Fountain View Drive #200, Houston, TX 77057-4832, USA; 2 Department of Anesthesiology, University General Hospital, Houston, TX 77030, USA

## Abstract

**Introduction:**

Occipital peripheral nerve stimulation is an interventional pain management therapy that provides beneficial results in the treatment of refractory chronic occipital neuralgia. Herein we present a first-of-its-kind case study of a patient with neurofibromatosis type 1 and bilateral occipital neuralgia treated with occipital peripheral nerve stimulation.

**Case presentation:**

A 42-year-old Caucasian woman presented with bilateral occipital neuralgia refractory to various conventional treatments, and she was referred for possible treatment with occipital peripheral nerve stimulation. She was found to be a suitable candidate for the procedure, and she underwent implantation of two octapolar stimulating leads and a rechargeable, programmable, implantable generator. The intensity, severity, and frequency of her symptoms resolved by more than 80%, but an infection developed at the implantation site two months after the procedure that required explantation and reimplantation of new stimulating leads three months later. To date she continues to experience symptom resolution of more than 60%.

**Conclusion:**

These results demonstrate the significance of peripheral nerve stimulation in the management of refractory occipital neuralgias in patients with neurofibromatosis type 1 and the possible role of neurofibromata in the development of occipital neuralgia in these patients.

## Introduction

Chronic daily headache (CDH) syndromes represent a major health issue worldwide in terms of lost workdays and revenue [1-3]. Diagnoses include migraine, atypical migraine, cluster headaches, transformed migraines, cervicogenic headaches, occipital and facial hemicranias, or any combination of these diagnoses. Many of the patients who experience these syndromes become totally disabled after conservative and pharmacological treatments fail to relieve their symptoms [4,5].

Occipital neuralgia is described by the National Institute of Neurological Diseases and Stroke as a "distinct type of headache characterized by piercing, throbbing, or electric-shock-like chronic pain in the upper neck, back of the head, and behind the ears, usually on one side" [6]. Typically, the pain of occipital neuralgia begins at the base of the head and spreads upward within the distribution of the greater and lesser occipital nerves. Characteristically, it is neuropathic, with paroxysmal episodes of shooting electric shock-like symptoms. Although the symptom etiology includes trauma, infection, and surgery, most patients with occipital neuralgia have idiopathic etiologies of their pain [7].

Neurofibromatosis type 1 (NF-1) is a possible but undocumented idiopathic etiology of occipital neuralgia. This human genetic disease, which is caused by mutations of the NF-1 tumor suppressor gene, has an incidence of about one in every 2500 live births and has a high rate of spontaneous mutations [8]. The characteristic feature of NF-1 is neurofibromata, which are complex lesions of the peripheral nervous system [8]. These lesions are composed of abnormal local cells, including Schwann cells, endothelial cells, fibroblasts, and a large number of infiltrating inflammatory mast cells [8,9]. They can cause a variety of symptoms when they invade surrounding tissues. Other characteristics of NF-1 are flat, pigmented lesions of the skin (café au lait spots), axillary freckles, pigmented iris hamartomas (Lisch nodules), and a variety of central nervous system manifestations, such as optic nerve tumors, unidentified bright objects in the visual field, and neurofibromas of the spinal nerve roots (schwannomas) [10]. Although headaches are very common in patients with NF-1, the specific diagnosis of occipital neuralgia is not [11-13].

The initial treatment for both CDH syndromes and occipital neuralgia is pharmacologic and is focused on symptom relief [14]. Patients whose symptoms do not respond to this initial therapy are treated secondarily with occipital nerve blockade [15], radiofrequency ablation [16], botulinum toxin A injections [17,18], surgical decompression [19], and occipital peripheral nerve stimulation (OPNS) [7,20-23]. OPNS involves the placement of trial peripheral nerve-stimulating electrodes over the occipital nerves. If the prerequisite dermatomal paresthesia is achieved, then pain relief as a result of permanent implantation has been reported to be as high as 80% [7,20-23]. In this report, we present the case of a woman with NF-1 and bilateral occipital neuralgia who experienced pain relief after OPNS.

## Case presentation

### Patient history

A 42-year-old Caucasian woman was referred to our hospital for pain management by a neurologist specializing in the treatment of daily headaches. She had experienced daily intractable headaches since age 18 years. She also had chronic bilateral occipital neuralgia on the basis of the diagnostic criteria outlined in the second edition of The International Classification of Headache Disorders [24]. Her occipital neuralgia persisted for more than 15 days monthly and was distributed throughout the greater occipital nerves, beginning in the occipital region and radiating upward to the top of the head. When the occipital neuralgia occurred, her occipital area became very tender to palpation. Complete alleviation of her pain had been achieved for a limited time with diagnostic bilateral greater occipital nerve blocks.

Her medical history included NF-1, which was first diagnosed in childhood. Several neurofibromas had been removed from her sacrum 10 years previously, as well as many from her upper extremities. She also had had problems with depression, anxiety, alcohol consumption, and smoking. She has been a housewife throughout her adult life. With regard to her family medical history, her mother had died at 68 years of age as a result of heart disease, and her father was alive at 72 years of age with a history of cancer. An older sister has rheumatoid arthritis but not NF-1.

Before her referral to our service, she had undergone extensive medical management with biofeedback training, physical therapy, massage, acupuncture, and pharmacological management with narcotic and non-narcotic pain medications. Her medications included sustained-release morphine (30 mg every 12 hours), hydrocodone and acetaminophen (10 mg and 325 mg, respectively, every four to six hours), and pregabaline (75 mg every eight hours). More recently, she had undergone three greater occipital nerve blocks that resulted in complete pain resolution that lasted from two to three days. Because she required an ever-increasing dose of morphine for pain relief, and because she had responded to the occipital nerve blocks, she was considered to be a good candidate for OPNS.

### Trial procedure

At her baseline office visit, the patient underwent a disability and quality-of-life assessment by completing a series of questionnaires (see "Quality-of-life assessment" section below) and was found to be a suitable candidate for a trial of OPNS. After the risks and benefits of the procedure were discussed with the patient and her informed consent was obtained, the trial of OPNS was carried out in October 2008 by using two percutaneous eight-contact leads (Octrode; St Jude Medical Neuromodulation Division, Plano, TX, USA). After a week-long successful trial with more than 80% symptom improvement, the patient was deemed a suitable candidate for permanent implantation and she underwent implantation of two permanent percutaneous eight-contact leads (Octrode) and a conventional implantable pulse generator (IPG) (Genesis; St Jude Medical Neuromodulation Division).

### Permanent implantation procedure

**Figure 1:**
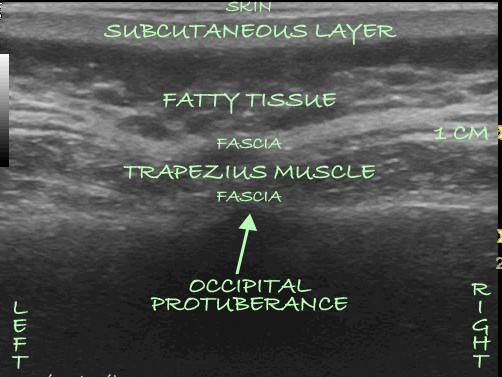
**An ultrasound image obtained with a linear transducer placed over the greater occipital protuberance**. The anatomical layers are identified sonographically starting from the surface and progressing toward the deeper layers.

**Figure 2:**
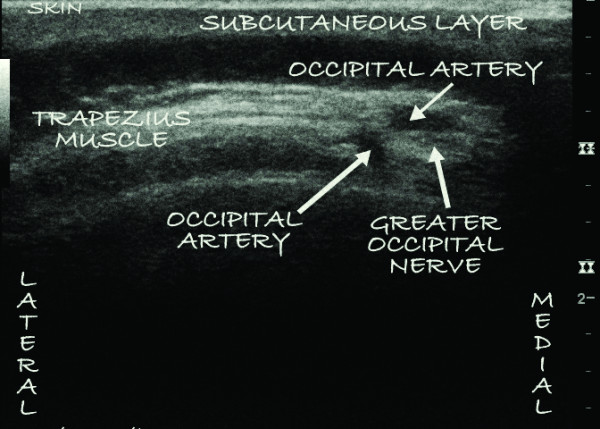
**An ultrasound image of the left occipital nerve as well as two divisions of the greater occipital artery as they pierce the substance of the trapezious muscle 1.0 cm to 1.2 cm from the skin**.

**Figure 3:**
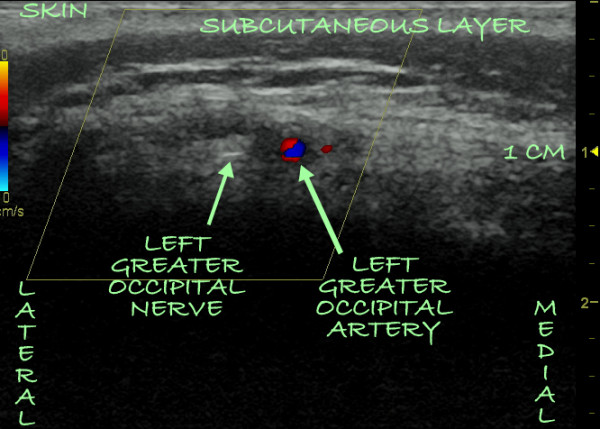
**A Doppler ultrasound image of the left occipital nerve and artery as they pierce the substance of the trapezius muscle side-by-side 1 cm from the skin**.

**Figure 4:**
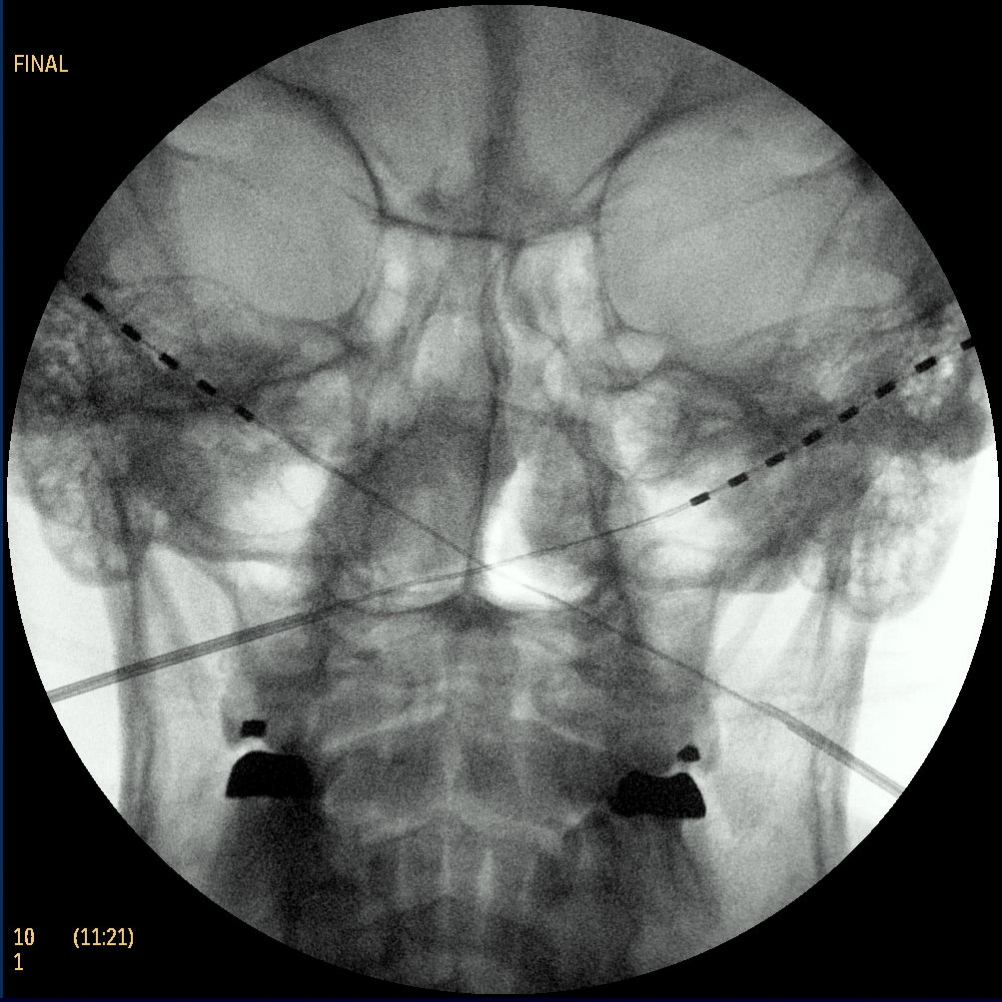
**Placement of two octapolar stimulating array leads over the bilateral occipital areas**. Placement was carried out with ultrasound guidance and fluoroscopy. The C1-C2 interspace, which serves as a key fluoroscopic landmark for occipital peripheral nerve stimulation, and the dens are easily identifiable.

On the day of the procedure, which was carried out in an operating room, a slow intravenous infusion of 2 g of cefazolin was started, and the patient was placed in a prone position with pillows under her chest to augment neck flexion. Monitored anesthesia was administered by using intravenous fentanyl and midazolam at a level that allowed the patient to be comfortable but able to interact with medical personnel throughout the procedure. The patient's hair was shaved below a line connecting the external occipital protuberance to the mastoid processes, and her skin was treated with chlorhexidine. A sterilely draped C-arm was introduced to obtain a true anteroposterior image of the cervical spine at the C1-C2 interspace, and the overlying skin was marked with a sterile marker. Thereafter a portable ultrasound with a sterile linear array transducer of 5 MHz to 13 MHz frequency was placed to obtain images of the bilateral occipital fossae and the bilateral greater occipital nerves and arteries. The ultrasound probe was first placed at the midline just below the external occipital protuberance (Figure 1). The probe was slowly advanced laterally at the same level until the greater occipital artery and nerve were visualized as two distinct structures: the artery as a hypoechogenic oval structure and the nerve as a hyperechogenic structure (Figures 2 and 3). The nerve could be traced from its exiting trunk into two distinct divisions within the substance of the trapezious muscle. The artery was identified by using Doppler ultrasound (Figure 2). The locations of the nerve and the artery were marked bilaterally on the skin with a sterile marker. The depths of both the artery and the nerve were found to be consistent at 1.0 cm to 1.2 cm from the skin surface. The skin overlying the greater occipital protuberance was injected with 2 ml or 3 ml of 1% lidocaine as a local anesthetic, and the stimulating electrodes were introduced through a 14-gauge introducer needle (0.5 cm to 0.7 cm below the skin surface) in a mediolateral position. Positioning was guided by the skin markings and was verified by fluoroscopy to complement the ultrasonographic images (Figure 4). The electrodes were tested intra-operatively by confirming adequate dermatomal paresthesia within the occipital nerve distribution. Implantation of the electrodes was performed by creating a mid-line subcutaneous pocket at the site of needle insertion. The implantable, programmable, rechargeable generator was permanently implanted in a subcutaneous pocket area in the left buttock. For the implantation, a local anesthetic (0.25% bupivacaine with epinephrine 1:200,000 to a total of 20 ml) was used for skin and tissue infiltration.

### Re-implantation of the peripheral nerve-stimulating leads

**Figure 5:**
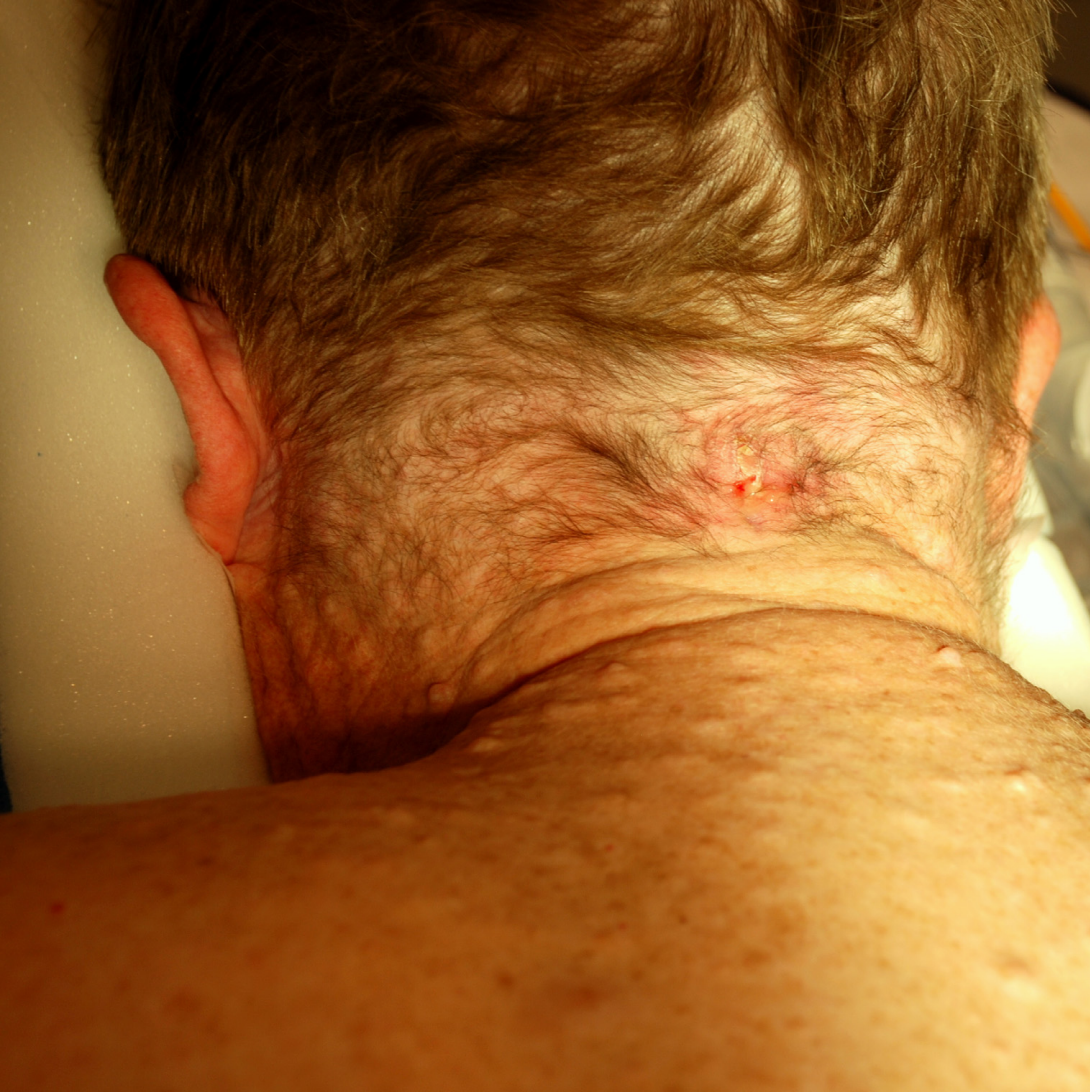
**Infected area over the site of the initial occipital implantation at mid-line**. Multiple neurofibromata are seen over the patient's back and neck area.

Two months after the implantation procedure, she developed an infection over the occipital implantation area, and the leads and the IPG were removed (Figure 5). Bacterial cultures were not obtained during the removal procedure. Her recovery from the procedure was uneventful. After the infection resolved with antibiotic treatment, she underwent re-implantation of two permanent octapolar leads and a new IPG (Eon Mini; St Jude Medical Neuromodulation Division) in March 2009 without additional complications.

### Quality-of-life assessment

The patient's pain level and quality of life were assessed at baseline and again at one, three, and six months after implantation. The questionnaires used in these assessments were the short form McGill Pain Questionnaire [25], the Visual Analogue Scale (VAS) [26], the Oswestry Disability Questionnaire [27], and the SF-36 Health Survey [28]. The data gathered from these questionnaires were plotted for comparison. Qualitative data were also collected from the patient's medical record.

After the trial implantation, the patient experienced nearly an 80% reduction in headache severity. Although the initial implantation procedure was complicated by infection in the implantation site and the patient underwent reimplantation, she has experienced sustained benefit from the treatment and remains infection-free. Over time, the severity, frequency, and duration of her headaches have improved by more than 60%. She continued to use hydrocodone and pregabaline, but was able to discontinue use of morphine. All outcome measures of pain and quality of life were positively affected by the treatment. She reported improvement in her quality of life, which she characterized as "being more active and enjoying life," being able to exercise, working for longer hours, and having improved mood.

## Discussion

Neurofibromatosis is an autosomal dominant, genetically inherited disease first described in 1882 by the German pathologist Friedrich Daniel von Recklinghausen. It belongs to the family of phakomatoses and is subcategorized into two types: NF-1 (von Recklinghausen's disease) and NF-2 (bilateral acoustic neurofibromatosis). Our patient had classic NF-1, which is characterized predominately by neurofibromas of the peripheral nervous system [8].

According to the National Institutes of Health, a definitive diagnosis of NF-1 is made when two of seven cardinal clinical features of the disease are present [29]: (1) six or more café au lait macules that measure >5 mm in the greatest diameter in prepubertal individuals and >15 mm in the greatest diameter in postpubertal individuals; (2) two or more neurofibromas of any type or one plexiform neurofibroma; (3) freckles in the axillary or inguinal region; (4) optic nerve glioma; (5) two or more iris hamartomas (Lisch nodules); (6) a distinctive osseous lesion, such as sphenoid dysplasia or thinning of the long-bone cortex with or without pseudoarthrosis; and (7) a first-degree relative with NF-1 diagnosed on the basis of the preceding six criteria.

There are many complications of NF-1, including chronic hypertension, pheochromocytomas, brain tumors, malignant peripheral nerve tumors, and a high incidence of learning disabilities [10]. The most significant complications are dermal and plexiform neurofibromas, malignant peripheral nerve sheath tumors, and other malignant tumors [10,11]. The known mechanisms by which tumor cells evade detection by the human immune system are thought to play a role in the progression to malignancy in patients with NF-1 [30].

Chronic headaches are among the most common neurological manifestations of NF-1. In a hospital-based series of 158 patients with NF-1 [11], headache was one of the most common neurological manifestations. Twenty-eight (28%) of the patients were found to have chronic idiopathic headaches and migraines. Although headaches are common in patients with NF-1, the frequency has varied between studies. In one study of 181 patients with NF-1 [12], headache was present in 55 patients (30%). This frequency was not statistically significantly different from that found in the general population, leading the study investigators to conclude that headache is not a specific feature of NF-1. This conclusion, however, was contradicted by the findings of another study [13] that comprised 132 patients with NF-1. Eighty-one (45%) of these patients were found to have headaches, a frequency that led the study investigators to conclude that patients with NF-1 are at greater risk for headaches than the general population. Another interesting finding of the study was that 38 (47%) of the 81 patients had recurrent headaches [13].

Although an association between NF-1 and distinct neurological syndromes, such as the Arnold-Chiari I malformation, has been reported [31], no association has been found between NF-1 and occipital neuralgia. The most common skull manifestations of NF-1 involve the orbit, with very few reports of occipital defects in patients with NF-1 [32]. Such occipital defects have been found in a 54-year-old woman with a massive plexiform neurofibroma that extended from the auricular region to the shoulder and was associated with large left occipital and left petrous bone defects [32].

No neurofibromatous lesions were visualized sonographically during the placement of the stimulating leads in our patient, but multiple small neurofibromata were found during creation of the implant pocket at the posterior neck site, and they were dissected. A subsequent computed tomographic scan of her head revealed subcutaneous nodules in the tissue surrounding the stimulating leads in both occipital areas, which were consistent with neurofibromas. The histological diagnosis of these nodules is unknown, since the nodules were not sent for pathological analysis. The clinical significance of these nodules is also unknown, since they could interfere with the surrounding branches of the greater and lesser occipital nerves and create most of the symptomatology reported by our patient.

Of interest is the fact that during ultrasound guidance for OPNS, the greater occipital nerve was visualized both medially (Figure 2) and laterally (Figure 3) to the greater occipital artery. The variable course of the greater occipital nerve as it relates to the greater occipital artery, as well as the ability of ultrasonography to accurately identify both structures, makes a strong argument for the utilization of ultrasound guidance.

Only one other study has reported the use of neurostimulation as a treatment for neurofibromatosis [11]. In that study, three patients who had headaches that were refractory to conventional treatment were treated, respectively, with cutaneous neurostimulation, spinal cord neurostimulation, and cortical stimulation. Our patient responded very well to OPNS, achieving an 80% reduction in symptoms initially and a persistent >60% reduction at 10 months after electrode implantation. This outcome is in agreement with the outcomes of OPNS in studies of patients without NF-1 and is independent of the etiology of occipital neuralgia [7,20-23].

Our patient's recovery from implantation of the leads was complicated with infection at the implantation site approximately two months after the initial implantation. It is unusual for any implant to become infected after such a prolonged period and could possibly be a consequence of NF-1, since patients with neurofibromatosis are thought to be prone to infections because of a compromised immune system resulting from mast cell infiltration around neurofibromata [31].

## Conclusion

In conclusion, we report the successful treatment of chronic occipital neuralgia in a 42-year-old Caucasian woman with NF-1. Additional case reports or case series would give us a better understanding of the relationship between occipital neuralgia and NF-1 as well as the efficacy of OPNS in the treatment of occipital neuralgia in patients with this genetic disorder.

## Consent

Written informed consent was obtained from the patient for publication of this case report and any accompanying images. A copy of the written consent is available for review by the Editor-in-Chief of this journal.

## Competing interests

The authors declare that they have no competing interests.

## Authors' contributions

IMS performed all procedures, obtained the patient's written informed consent to publish the report, conducted the follow-up examinations, analyzed and interpreted the patient data, and wrote and edited the manuscript. OC was a major contributor to reviewing and editing the manuscript. EDS contributed to the review and editing of the manuscript. All authors read and approved the final manuscript.
